# BETter together: exploiting BRD4-functions in transcription to inform rational combinations

**DOI:** 10.18632/oncoscience.415

**Published:** 2018-06-23

**Authors:** Fabio Savarese, Norbert Kraut

**Affiliations:** Boehringer Ingelheim RCV GmbH & Co KG, Vienna, Austria

**Keywords:** BRD4, BET inhibitors, CDK9, PARP, drug combinations

The identification of inhibitors of the BET family of bromodomain proteins (BETi) has fueled our knowledge about functions of their targets, the transcriptional regulators BRD2/3/4 and T, and this class of antitumor agents holds promise in clinical development [1]. However, even in pre-clinical models derived from hematological malignancies, which are in general more sensitive to BETi than those originating from solid cancers [2], use of BETi as single agents rarely results in strong apoptosis induction or tumor regressions at tolerated doses [1], underscoring the requirement for rational combination approaches.

Correlating BRD4-chromatin binding to gene expression upon treatment with the BETi BI 894999, Gerlach et al. demonstrated that intermediate levels of the compound only incompletely evict BRD4 from chromatin, which may limit its antitumor activity [1]. Regulatory elements, highly bound by BRD4, are enriched for lineage-specific transcription factor binding sites, raising the possibility that the activity of these (transacting) factors could limit the effects of BET-inhibition. The search for additional transcriptional regulators, potentially co-operating with BRD4, revealed that CDK9 is specifically co-localized with BRD4 both at promoters as well as at enhancers ([1] and unpublished data). Indeed, the combination of BI 894999 with various CDK9 inhibitors yielded synergistic effects on limiting cell proliferation, on inducing apoptosis and on total levels of the transcriptional elongation mark p-Ser2 POL-II at doses inefficacious as monotherapies [1].

At the chromatin level, the combination of BETi and CDK9i resulted in global inhibition of transcriptional elongation, as measured by genome-wide POL-II localization and sequencing of *de novo* transcripts (SLAM-seq; [1, 3]). This finding has various important implications: therapeutically, the question whether globally arresting transcriptional elongation is a valid concept will require a better mechanistic understanding of transcriptional dependencies in cancer, and cautious dosing and scheduling in early clinical trials will be important to address potential overlapping toxicities, including myelosuppression. Mechanistically, a major unresolved topic is the understanding of the precise role(s) which BRD4 plays in the regulation of gene expression. Treatment with high levels of BI 894999 or JQ1 leads to almost complete loss of BRD4 binding to chromatin and global inhibition of transcriptional elongation [1, 3]. In contrast, more therapeutically relevant concentrations of the BET inhibitors cause de-regulation of only a few hundred hypersensitive genes, including *MYC*. In this regard, it is noteworthy that mutating the bromodomain of BRD4 only affected its function in transcriptional elongation, but not its localization at promoters/transcriptional start sites (TSS) [4]. Likewise, the POL-II profile of BI 894999-regulated genes, like *MYC*, is perturbed by compound treatment in gene bodies and at the 3′ end of genes, but not at the TSSs ([1] and unpublished data). This suggests that monotherapy with BET inhibitors may affect only genes whose elongation is driven from distal regulatory elements, including enhancers, where BET inhibition is known to be particularly effective in antagonizing binding of BRD4 to chromatin [1, 4]. Genes, characterized by BRD4-binding solely to promoters/TSS, will not be affected by BET inhibition and are expected to require combination with CDK9 inhibition to stall transcriptional elongation. Notably, the effects observed via targeted BRD4 protein degradation, either by PROTACs or by an inducible system are mechanistically similar to the consequences of combined BET/CDK9-inhibition [3, 5]. We find it striking that BRD4-degradation has such a drastic effect on transcriptional elongation without affecting CDK9 recruitment [5]. Hence one can speculate that CDK9 may globally regulate BRD4's proposed function as an elongation factor and histone chaperone [4].

Why is this interplay between core regulators of transcription and transcriptional elongation so intriguing? The search for combination partners for BET inhibitors has recently led to the discovery of synergistic responses to PARP inhibitors [6]. BET-dependent transcriptional regulation of key effectors of DNA damage signaling like *CtIP* may explain much of the synergy between BETi and PARPi [6]. Interestingly, in addition to its well established roles in DNA damage repair, PARP1 itself was shown to be a regulator of transcriptional elongation [7]. PARylation of the negative elongation factor NELF downstream of CDK9 facilitates elongation and its inhibition led to increased POL-II pausing. When BETi and PARPi are combined, it will thus be important to examine the concomitant roles of BRD4 and PARP1 in the regulation of transcriptional elongation in addition to PARP1 functions in DNA repair.

This complexity underlying transcriptional plasticity and re-wiring following BET inhibition was also highlighted by another recent study [8]. Prostate cancer cells made resistant to BETi by extended JQ1 treatment were exquisitely sensitive to either CDK9 or PARP1 inhibition, unlike their parental cells. The acquired sensitivity to CDK9i was explained by CDK9-dependent phosphorylation and activation of the androgen receptor in BETi-resistant cells. Acquired PARPi-sensitivity was linked to epigenetic silencing of DNA damage response genes no longer dependent on BRD4, resulting in increased DNA damage. This suggests that not only the combination of BETi with CDK9i or PARP1i might be of therapeutic value, but also their scheduling holds promise.

Together, these recent reports support that rational combinations with either CDK9i or PARPi have the potential to increase the activity of BETi in a range of different cancers. New opportunities for BET inhibitors as “backbones” for combination therapies will likely continue to emerge, either by modulation of specific genes or via global effects on transcription [1-3, 6]. Further studies of the molecular consequences of acute and persistent BET inhibition will be necessary to better guide prioritization of drug combinations and schedules in order to unlock their optimal therapeutic potential.
